# Generalized contrastive PCA is equivalent to generalized eigendecomposition

**DOI:** 10.1371/journal.pcbi.1013555

**Published:** 2025-10-30

**Authors:** Joshua P. Woller, David Menrath, Alireza Gharabaghi

**Affiliations:** 1 Institute for Neuromodulation and Neurotechnology, University Hospital and University of Tübingen, Tübingen, Baden-Württemberg, Germany; 2 German Center for Mental Health (DZPG), Tübingen, Baden-Württemberg, Germany; 3 Max Planck Institute for Biological Cybernetics, Tübingen, Baden-Württemberg, Germany; 4 Center for Digital Health, Tübingen, Baden-Württemberg, Germany; 5 Center for Bionic Intelligence Tübingen Stuttgart (BITS), Tübingen, Baden-Württemberg, Germany; 6 Max Planck-University of Toronto Centre for Neural Science & Technology (MPUTC), Canada/Germany; Universitatsklinikum Hamburg-Eppendorf, GERMANY

***PLOS Computational Biology*** recently published an article by de Oliveira and colleagues introducing a novel data decomposition method that enables explicit contrasts between experimental conditions, demonstrating its applicability across a range of biological datasets [1]. We fully agree with their call for improved multivariate analyses and share the authors’ critique of the limitations of contrastive PCA (cPCA; [2]), especially the reliance on hyperparameters. To address this, the authors introduce a novel contrastive decomposition via an eigendecomposition of pairs of contrast matrices, called generalized contrastive PCA (gcPCA).

However, our analysis of the gcPCA algorithm suggests that the core approach of gcPCA is mathematically equivalent to solving a generalized eigenvalue problem, which is a well known approach in linear algebra to perform a contrastive matrix decomposition. Here, we aim to position gcPCA within the broader framework of generalized eigenvalue decomposition (GED), highlighting its theoretical foundation and connection to established methods. Toward the end, we will provide a mathematical derivation of this equivalence.

The generalized eigenproblem can be formulated and solved in multiple ways, making it non-trivial to recognize that it underpins a broad range of seemingly distinct algorithms. It is for example used in linear discriminant analysis (LDA) or canonical correlation analysis (CCA). An excellent overview of its background and applications particularly in neuroscience, e.g. to decompose task-evoked activity with respect to baseline activity, is provided by Cohen [3]. GED has been used to remove stimulation-evoked activity from oscillatory background signals [8], or to extract spatial filters that maximize theta-band activity [9]. Further, GED has been used to remove specific neurostimulation artifacts from EEG data [10].

Closer to gcPCA, discriminative PCA (dPCA) [7] has previously been proposed as a GED-based improvement of cPCA that also eliminates the need for hyperparameters. This shows that GED is the basis of a whole family of methods.

In parallel to other GED-based methods we hence argue that gcPCA constitutes a supervised method, since it relies on the definition of two matrices derived from covariances of different data segments. Aggregating data into separate matrices based on a researcher-defined criterion effectively provides the algorithm with labeled data, even if labels are not explicitly passed along. By construction, such methods should not be considered unsupervised, in contrast to approaches such as principal component analysis (PCA) or independent component analysis (ICA).

Furthermore, we would like to highlight a key advantage of gcPCA: the extracted components are not required to be orthogonal in feature space, a property shared with other GED-derived methods, and notably ICA. Non-orthogonality in feature space is a key strength compared to standard PCA. Orthogonalization restricts subsequent components to subspaces orthogonal to previously extracted ones; this makes it difficult to interpret later principal components independent of earlier ones. In this light, a stronger mathematical rationale for orthogonal gcPCA, especially how regular gcPCs relate to their orthogonalized counterparts, would clarify whether orthogonality aids or hinders analysis. For instance, alternating extraction of high- and low-eigenvalue components may introduce bias, particularly if low-eigenvalue noise components are allowed to restrict the subspace into which high-eigenvalue signals are then projected. The degree to which extraction order influences orthogonal gcPCA outcomes remains unclear. While such drawbacks of the orthogonalization are not evident in S3 Fig of [1], standard gcPCs in this example already appear to be close to orthogonality, obscuring possible detrimental effects of the method.

Departing from those general observations, we briefly review the original algorithm, and demonstrate its equivalence to a generalized eigenproblem.

Let ${𝐂}_{a},{𝐂}_{b}\in {\mathbb{R}}^{n\times n}$ be covariance matrices, and let $𝐀,𝐁\in {\mathbb{R}}^{n\times n}$ be matrices defined as linear compositions of ${𝐂}_{a}$ and ${𝐂}_{b}$ (or 𝐁=𝐈 for cPCA). The authors define various versions of gcPCA, with varying values of 𝐀,𝐁 (see Table 1):

**Table 1:** Variants of contrastive or generalized contrastive PCA (gcPCA), defined by different choices of matrices A and B.

Version	Definition
1 (cPCA, [2])	$𝐀={𝐂}_{a}-\alpha {𝐂}_{b},\;𝐁=𝐈$
2 (gcPCA)	$𝐀={𝐂}_{a},\;𝐁={𝐂}_{b}$
3 (gcPCA)	$𝐀={𝐂}_{a}-{𝐂}_{b},\;𝐁={𝐂}_{b}$
4 (gcPCA)	$𝐀={𝐂}_{a}-{𝐂}_{b},\;𝐁={𝐂}_{a}+{𝐂}_{b}$

The authors use the following optimization criterion:

$$\underset{𝐱:{𝐱}^{⊤}𝐱=1}{\arg\max}\;\frac{{𝐱}^{⊤}𝐀𝐱}{{𝐱}^{⊤}𝐁𝐱}$$ (1)

To maximize this contrast (1) between matrices 𝐀,𝐁, the authors solve the following eigenproblem:

$${\sqrt{𝐁}}^{-1}𝐀{\sqrt{𝐁}}^{-1}𝐮=\lambda 𝐮$$ (2)

where $\lambda_{i},{𝐮}_{i}$ are the eigenvalues and eigenvectors, respectively.

We may identify expression (1) as the *generalized Rayleigh coefficient*:

$$R_{A,B}(𝐱)=\frac{{𝐱}^{*}𝐀𝐱}{{𝐱}^{*}𝐁𝐱}$$ (3)

The Rayleigh coefficient is used to solve standard (assuming 𝐁=𝐈) and generalized eigenvalue problems.

The generalized eigenproblem can be formulated as follows:

𝐀𝐱=λ𝐁𝐱 (4)

where $\lambda_{i},{𝐱}_{i}$ are the generalized eigenvalues and eigenvectors, respectively. Decomposing a matrix like this is called a generalized eigendecomposition (GED) [3].

Solvers for this are implemented in numerical libraries such as LAPACK, and scientific computing libraries such as MATLAB or scipy, i.e. using eig(A,B) and linalg.eig(A,B), respectively. We have tested that these solvers produce equivalent solutions to the gcPCA methods in Table 1.

In the following, we will show that (2) is a generalized eigenproblem (4), up to a rotation of the eigenvectors by ${\sqrt{𝐁}}^{-1}$, which is also later applied in the provided gcPCA software package (https://github.com/SjulsonLab/generalized_contrastive_PCA).

The eigenproblem to maximize (1) is formulated as (2):


$${\sqrt{𝐁}}^{-1}𝐀{\sqrt{𝐁}}^{-1}𝐮=\lambda 𝐮\;(2)$$

This can be reformulated as follows:

$$𝐀{\sqrt{𝐁}}^{-1}𝐮=\lambda \sqrt{𝐁}𝐮\;\text{(Left-multiplied by }\sqrt{𝐁}\text{)}$$ (5)

$$𝐀{\sqrt{𝐁}}^{-1}\sqrt{𝐁}𝐱=\lambda \sqrt{𝐁}\sqrt{𝐁}𝐱\;\text{(Substituting }𝐮=\sqrt{𝐁}𝐱\text{)}$$ (6)

which simplifies to:

𝐀𝐱=λ𝐁𝐱, (7)

which is the generalized eigenproblem (4).

The eigenvalues of (2) and (4) for a given matrix pair (𝐀,𝐁) are identical. The corresponding eigenvectors **u** (2) and **x** (4) are related by the transforms


$$𝐱={\sqrt{𝐁}}^{-1}𝐮\;\text{and}\;𝐮=\sqrt{𝐁}𝐱.$$

For invertible **B**, (4) can be reduced to the following standard eigenproblem:

$$({𝐁}^{-1}𝐀)𝐱=\lambda 𝐱$$ (8)

A thorough mathematical and computational overview of generalized eigenproblems is given by Ghojogh and colleagues [4]. Given the connection between gcPCA and GED, it may further be helpful to consider how sparse gcPCA relates to sparse GED [5, 6]. This could offer insight into theoretical links and efficient algorithms.

Placing gcPCA within this broader methodological context of generalized eigenvalue decomposition provides a better understanding of its practical value and interpretive strengths. The study by de Oliveira and colleagues underlines the value of supervised contrastive decomposition techniques for biological data. Their approach, based on GED, produces interpretable components and shows substantial improvements over conventional PCA.
